# Heterotic grouping of provitamin A-enriched maize inbred lines for increased provitamin A content in hybrids

**DOI:** 10.1186/s12863-023-01156-z

**Published:** 2023-09-27

**Authors:** Abdoul-Raouf Sayadi Maazou, Melaku Gedil, Victor O. Adetimirin, Nnanna Unachukwu, Wende Mengesha, Silvestro Meseka, Abebe Menkir

**Affiliations:** 1 Pan African University Life and Earth Sciences Institute (including Health and Agriculture), University of Ibadan, Ibadan, 200284 Nigeria; 2 International Institute of Tropical Agriculture (IITA), PMB 5320, Ibadan, 200001 Nigeria; 3 Department of Crop and Horticultural Sciences, University of Ibadan, Ibadan, 200284 Nigeria

**Keywords:** Heterotic groups, Genetic diversity, Maize, Provitamin A, Inbred lines, testers, DArTag markers

## Abstract

**Background:**

The establishment of heterotic groups of inbred lines is crucial for hybrid maize breeding programs. Currently, there is no information on the heterotic patterns of the Provitamin A (PVA) inbred lines developed in the maize improvement program of the International Institute of Tropical Agriculture (IITA) to form productive PVA enriched hybrids for areas affected by vitamin A deficiency. This study assessed the feasibility of classifying PVA-enriched inbred lines into heterotic groups based on PVA content without compromising grain yield in hybrids. Sixty PVA inbred lines were crossed to two testers representing two existing heterotic groups. The resulting 120 testcrosses hybrids were evaluated for two years at four locations in Nigeria.

**Results:**

The two testers effectively classified the inbred lines into two heterotic groups. The PVA-based general combining ability and specific combining ability (HSGCA) method assigned 31 and 27 PVA enriched maize inbred lines into HGB and HGA, respectively, leaving two inbred lines not assigned to any group. The yield-based HSGCA method classified 32 inbred lines into HGB and 28 inbred lines into HGA. Both PVA and yield-based heterotic grouping methods assigned more than 40% of the inbred lines into the same heterotic groups. Even though both PVA and yield-based heterotic grouping of the inbred lines differed from the clusters defined by the DArTag SNP markers, more than 40% of the inbred lines assigned to HGA were present in Cluster-1 and 60% of the inbred lines assigned to HGB were present in Cluster-3. Interestingly, the inbred lines assigned to the same heterotic groups based on PVA content and grain yield were distributed across the three Ward’s clusters. The PVA-based HSGCA was identified as the most effective heterotic grouping method for breeding programs working on PVA biofortification.

**Conclusions:**

Selecting PVA enriched maize inbred lines with diverse genetic backgrounds from the three marker-based clusters may facilitate the development of productive hybrids with high PVA content and for generating source populations to develop more vigorous maize inbred lines with much higher concentrations of PVA.

**Supplementary Information:**

The online version contains supplementary material available at 10.1186/s12863-023-01156-z.

## Background

The development of maize hybrids with enhanced concentrations of provitamin A (PVA), high yield potential, desirable agronomic traits and adaptation to a wide range of environmental conditions requires knowledge of the heterotic affinities of the parental inbred lines. The most commonly used methods to group inbred lines into heterotic groups is based on specific combining ability (SCA) effects of inbred lines and grain yields of hybrids [1–4]. Fan et al. [5] also proposed the use of both general combining ability (GCA) effects of each inbred line and the SCA effects of the inbred lines in cross combinations (HSGCA) with known testers to classify inbred lines into heterotic groups. However, these approaches have not been used to separate PVA enriched maize inbred lines into heterotic groups based on PVA concentrations using the line x tester mating design to optimize expressions of PVA content in hybrids without compromising grain yields.

Molecular markers have also been used to provide complementary information to the field-based classification of maize inbred lines into heterotic groups to maximize the expression of heterosis in hybrids [6–8]. Furthermore, the molecular-based grouping allows breeders to select divergent parental inbred lines within a heterotic group for recycling to develop new maize inbred lines with greater concentrations of PVA content and desirable agronomic and adaptive traits. Suwarno et al. [7] reported high expression of heterosis for PVA content by crossing parental inbred lines selected based on genetic distances estimated using molecular markers. Genetic distance-based heterotic grouping using simple sequence repeat (SSR) markers have also been effective for increased performance under drought and optimal conditions [9]. In a recent study, Abu et al. [10] obtained five clusters using single nucleotide polymorphism (SNP) markers for tropical maize inbred lines and predicted high levels of heterosis in crosses involving parents from these clusters.

The development and deployment of maize varieties with high levels of PVA carotenoids has been considered an important complementary approach for addressing Vitamin A Deficiency (VAD) in sub-Saharan Africa (SSA). The maize improvement program (MIP) at the International Institute of Tropical Agriculture (IITA) has thus developed several maize inbred lines with high levels of PVA by mining novel alleles for high β-carotene from temperate donor inbred lines [11]. However, the heterotic affinities of the temperate donor inbred lines to the recipient elite tropical inbred lines forming backcrosses that were sources of the PVA enriched maize inbred lines were not known. Therefore, assessing the genetic diversity and separating these elite PVA enriched maize inbred lines into heterotic groups based on molecular markers and PVA content will be important for identifying parents to maximize the expression of heterosis for PVA content in hybrids. At the same time, understanding classification of these inbred lines based on grain yields is also critical for developing hybrids combining high concentrations of PVA with superior agronomic performance. This study was, therefore, conducted to assess the feasibility of using nutrient-based grouping of PVA enriched maize inbred lines without adversely affecting yield-based grouping of the inbred lines to develop high yielding hybrids with high PVA content.

## Results

### Combined analysis of variance and testcross performance for grain yield and PVA content

In the combined analysis of variance (ANOVA), environment had significant effects on grain yield and PVA content (Table 1). The GCA effect of the PVA inbred lines and the two testers were also significant for grain yield and PVA content. Likewise, the SCA effects (line × tester) were significant for both PVA content and grain yield (Table 1). The line × tester × environment interaction was not significant for grain yield, but was significant for PVA content (Table 1).

**Table 1:** Mean squares for grain yield and Provitamin A from combined analysis of variance of testcrosses of 60 provitamin A maize inbred evaluated across eight environments in Nigeria

Source of variation	DF	Grain yield	Provitamin A
Environment	7	711,565,530**	909.02**
Hybrid (H)	123	7,278,902**	25.83**
Testcross	119	7,221,635**	25.14**
Line (GCA)	59	10,025,419**	45.35**
Tester (GCA)	1	21,472,764**	1498.54**
Line × Tester (SCA)	59	9,365,602**	5.94**
Hybrid × ENV	860	1,592,711**	3.25**
Line × ENV	413	1,961,929	5.17**
Tester × ENV	7	19,595,613**	85.09**
Line × Tester × Env	413	1,314,650	3.48**
Error	500	1,136,506	1.36
Repeatability		0.82	0.79
CV (%)		17.19	10.25

*GCA* = general combining ability; *SCA* = specific combining ability; *CV* = Coefficient of Variation

*, ** significant at probability < 0.05, 0.01 levels, respectively

Mean PVA content and grain yield of the inbred lines in crosses with the two testers and their SCA effects are presented in Tables S1 and S2. All the T1 and T2 testcrosses had similar or significantly higher PVA content relative to the cross between the two testers (T1 × T2) (Table S1). On the average, testcrosses involving T1 had 2.5 µg/g more PVA than the testcrosses involving T2. Testcrosses of T1 had PVA content varying from 8.7 to 21.4 µg/g, whereas testcrosses of T2 had PVA content varying from 6.6 to 17.0 µg/g (Table S1). In contrast, testcrosses of T2 produced 216 kg/ha more grain yield than those of T1 (Table S2). Mean grain yields varied from 3994 to 7906 kg/ha for testcrosses of T1 and from 4,206 a to 7,618 kg/ha for the testcrosses of T2. Amongst the 60 T1 testcrosses, 7 had significantly higher mean grain yields than the cross between the two testers (T1 × T2), whereas 8 T2 testcrosses of T2 produced significantly higher grain yields than the cross between testers (Table S2).

First, the PVA-based SCA effects were used to separate the PVA enriched maize inbred lines into two heterotic groups (HGA and HGB). This method assigned 32 PVA enriched maize inbred lines into HGB and 26 PVA enriched maize inbred lines into HGA (Table S1). Two inbred lines that showed no SCA effects for PVA content were not assigned to any heterotic group. The HSGCA method also assigned the same 31 and 27 inbred lines into HGB and HGA, respectively, with the remaining two inbred lines classified into HGA.

The yield-based SCA effects were also used to assign the PVA enriched maize inbred lines into heterotic groups (HGA and HGB). HGB consisted of 24 inbred lines while 24 other inbred lines were classified into the HGA heterotic group (Table S2). The remaining 12 inbred lines with less than 100 kg/ha SCA effects were not assigned to any of the two heterotic groups (Table S2). The HSGCA values for grain yield with each tester were also used to classify the inbred lines into heterotic groups (Table S2). This method assigned 32 inbred lines into HGB and 28 inbred lines into HGA. It is interesting to note that the 23 inbred lines that were classified into HGA by the SCA method were also placed into the same heterotic group by the HSGCA method. Also, both the SCA and the HSGCA methods classified 24 inbred lines into HGB.

PVA-based HSGCA grouping was compared with that of yield-based HSGCA grouping of the inbred lines to assess the similarity of the compositions of the two groups defined by the two approaches. Amongst the 27 PVA enriched maize inbred lines that were classified into HGA based on PVA-based HSGCA, 11 were also classified into HGA using yield-based HSGCA (Tables S1 and S2). In addition, 16 PVA enriched maize inbred lines that were assigned to HGB based on PVA-based HSGCA were also classified into HGB using yield-based HSGCA. However, there was no significant correlation between the PVA-based and yield-based heterotic grouping methods (Table 2).

**Table 2:** Spearman correlation coefficients (top) and P values (bottom) among heterotic grouping methods (n = 60)

	PVA-SCA	PVA-HSGCA	GY-SCA
PVA-HSGCA	0.97014		
	< 0.0001		
GY-SCA	-0.01229	0.03553	
	0.9258	0.7875	
GY-HSGCA	0.02863	0	0.53536
	0.8281	1	< 0.0001

*PVA-SCA* Provitamin A-based grouping using SCA effects,

*PVA-HSGCA* Provitamin A-based grouping using HSGCA effects,

GY-SCA Grain yield-based grouping using SCA effects,

GY-HSGCA Grain yield-based grouping using HSGCA effects

### DArTag markers-based grouping of PVA enriched maize inbred lines

A total of 1879 informative SNP markers used to assess the genetic diversity among the PVA enriched maize inbred lines were distributed across the 10 chromosomes with chromosome 5 having the highest number of markers (Fig. S1). Gene diversity varied from 0.10 to 0.50 with a mean of 0.37, while PIC values ranged from 0.09 to 0.38 with an average of 0.30. Major allele frequency varied from 0.50 to 0.95 with an average of 0.72 with the mean heterozygosity ranging from 0 to 0.19 with a mean of 0.09 (Fig. S1).

**Fig. 1:**
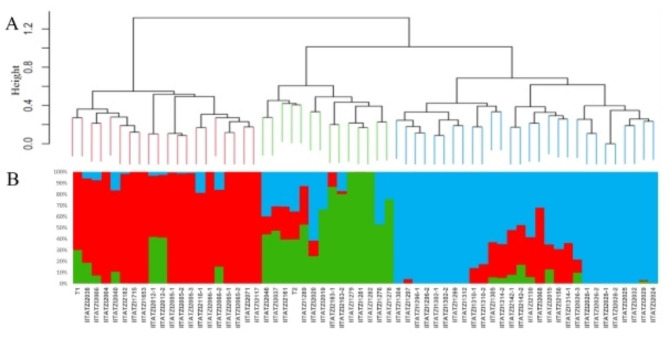
(**A**) Clustering of 60 PVA inbred lines and two testers using Ward’s method. (**B**) Estimated population structure of the inbred lines as revealed by the 1879 SNP markers for K = 3. Cluster 1, cluster 2, and cluster 3 are coloured with red, green, and blue, respectively

The Ward’s hierarchical cluster dendrogram grouped the 60 PVA inbred lines and the two testers into three main clusters (Fig. 1A). The first cluster consisted of 19 inbred lines and tester T1. Tester T2 and 13 inbred lines were grouped into the second cluster, with the remaining 28 inbred lines grouped in Cluster-3 (Fig. 1A). Nearly 41% of the PVA enriched maize inbred lines assigned to HGA were included in Cluster-1, whereas about 65% of the inbred lines assigned to HGB based on PVA content were included in Cluster-3. Also, about 57% of the PVA enriched maize inbred lines assigned to HGA based on grain yield were present in Cluster-1, while 69% of the inbred lines assigned to HGB were present in Cluster-3. It is interesting to note that the inbred lines that were assigned to the same heterotic group based on PVA content and grain yield were distributed across the three Ward’s clusters. The PVA enriched maize inbred lines included in Cluster-1 had an average PVA content of 15.7 µg/g in crosses with T1 and 12.0 µg/g in crosses with T2 (Table S3). The inbred lines in Cluster-1 also produced an average grain yield of 5238 kg/ha in crosses with T1 and 6463 kg/ha in crosses with T2. The PVA inbred lines grouped in Cluster-2 had an average PVA content of 13.2 µg/g in crosses with T1 and 9.9 µg/g in crosses with T2. These inbred lines showed an average grain yield of 6375 kg/ha in crosses with T1 and 6510 kg/ha in crosses with T2. The average PVA content of the inbred lines included in Cluster-3 13.6 µg/g in crosses with T1 and 10.8 µg/g in crosses with T2. The inbred lines in Cluster-3 produced an average grain yield of 6531 kg/ha in crosses with T1 and 6088 kg/ha in crosses with T2 (Table S3). The PVA content of the inbred lines was reported by Maazou et al. [12].

**Fig. 2:**
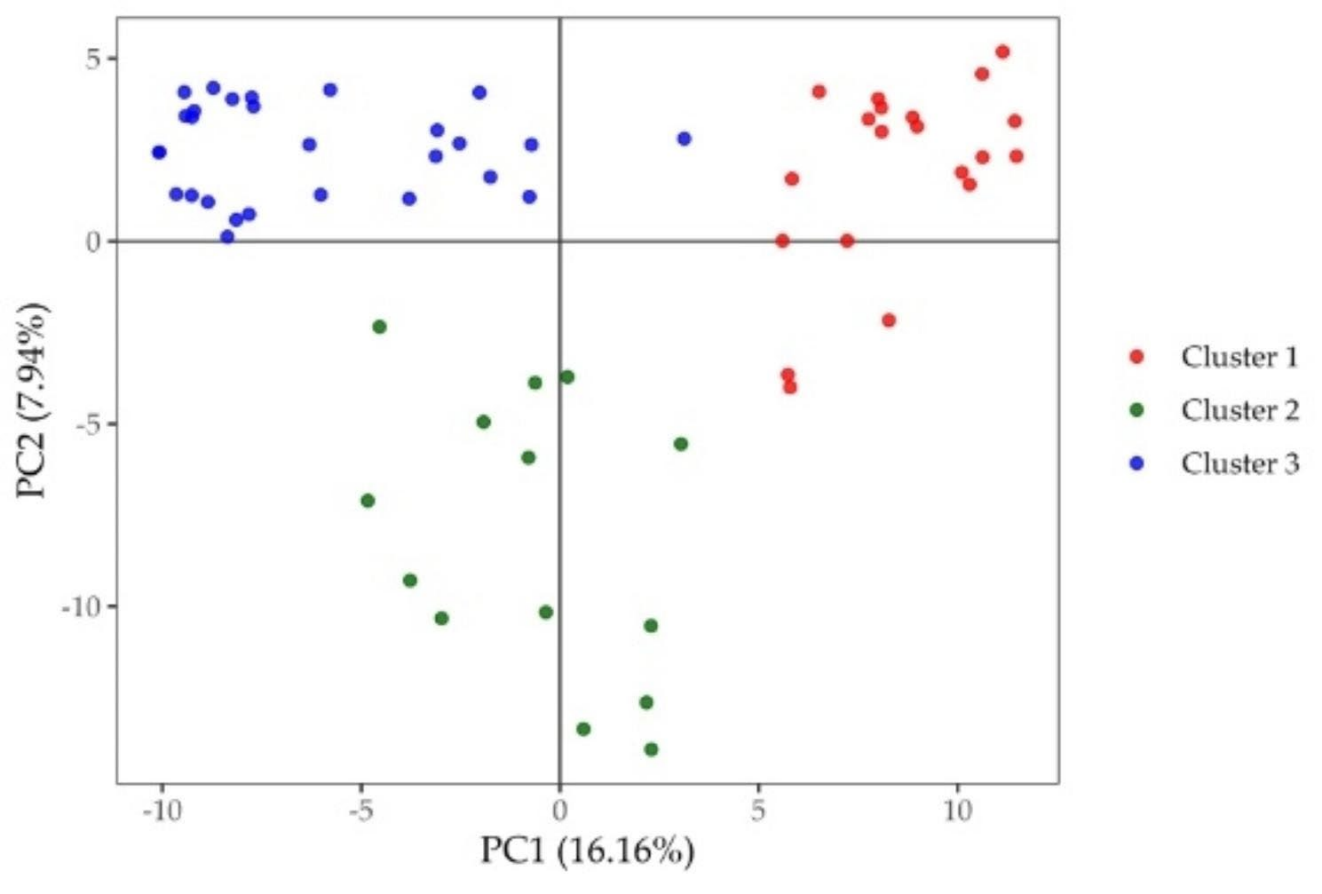
Principal Component Analysis (PCA) of the SNP data of the 60 PVA enriched maize inbred lines and the two testers

The results of genetic structure procedure also classified the inbred lines into three main clusters (Fig. 1B and Table S3). This analysis was consistent with the Ward’s cluster analysis in classifying the inbred lines into three main clusters with only 1 inbred line each belonging to Wards’ Cluster-2 and Cluster-3 assigned to different clusters based on structure analysis. Also, three inbred lines assigned to Ward’s Cluster-1, five inbred lines included in Ward’s Cluster-2 and three inbred lines included in Ward’s Cluster-3 with membership probabilities below 60% were assigned to a mixed group. The genetic structure and Ward’s cluster analyses were confirmed using principal component analyses of the SNP markers data, which separated the PVA enriched maize inbred lines into three groups (Fig. 2).

## Discussion

The development of maize hybrids with high concentrations of essential micronutrient, high yield potential, and desirable agronomic traits is a complementary approach to combat food and nutritional insecurity in developing countries [13, 14]. In a hybrid maize breeding program, knowledge of the heterotic patterns of parental inbred lines is essential to develop nutrient dense and productive hybrids. The present study was thus conducted to evaluating PVA enriched maize inbred lines in crosses with known testers for classifying them into heterotic groups using PVA content and grain yield. The PVA and non-PVA carotenoids contents and the agronomic performance for all testcrosses were reported by Maazou et al. [15]. The testers successfully separated 58 inbred lines into two heterotic groups using PVA content-based HSGCA effects. The PVA content-based HSGCA heterotic grouping was indeed the most effective method of heterotic grouping in the present study as it separated the highest number of inbred lines into different groups. Hybrids formed from crosses of PVA inbred lines representing the resulting HGA and HGB are expected to have higher expression of heterosis in PVA accumulation. Further classification of these inbred lines was made using yield-based HSGCA, which assigned 28 inbred lines into HGA and 32 inbred lines into HGB. Both PVA-based and grain yield-based heterotic grouping assigned more than 40% of the PVA enriched maize inbred lines into the same heterotic group. Selection of such inbred lines from the two heterotic groups as parents for crossing can then enhance PVA content and increase productivity in hybrids. The two inbred lines that were not classified into the existing heterotic groups could be further crossed with new testers representing the existing heterotic groups that have different genetic backgrounds from the testers used in the present study.

Maazou et al. [12] evaluated the same set of PVA enriched maize inbred lines for carotenoid composition and content and found that inbred lines with the highest level of PVA were included in Ward’s Cluster-1 and the remaining inbred lines with medium-to-high concentrations of PVA were assigned to Ward’s Cluster-2 and Cluster-3. As each of the two heterotic groups defined based on PVA-based HSGCA are represented by inbred lines classified into the three Ward’s clusters, high PVA enriched inbred lines within each heterotic group selected from the different Ward’s clusters can be used as parents to make bi-parental crosses for developing new maize inbred lines with much higher levels of PVA.

Considerable genetic diversity was found among the PVA enriched maize inbred lines. The average gene diversity of 0.37 observed in this study was higher than the 0.25 reported by Dao et al. [16], but lower than the 0.39 reported by Yang et al. [17]. The PIC value of 0.30 obtained in this study was also higher than the 0.28 and 0.29 reported by Zhang et al. [18] and Abu et al. [10], respectively. The DArTag marker-based clustering of the PVA enriched maize inbred lines was different from the PVA and yield-based grouping of the inbred lines. Most of the PVA enriched maize inbred lines classified into HGA using the yield-based HSGCA grouping method were present in Ward’s Cluster-1, whereas those assigned to HGB using the same method fell in Ward’s Cluster-3. As more than 40% of the PVA enriched inbred lines were consistently assigned to the same heterotic group using the two grouping methods, selection of such inbred lines from the two heterotic groups as parents may promote the development of hybrids combining high concentrations of PVA with high yield potential. Furthermore, selection of PVA enriched maize inbred lines from different molecular markers-based clusters within each heterotic group can facilitate the generation of source populations for developing new maize inbred lines with high PVA content and desirable agronomic features.

## Conclusions

The inbred line testers used in the present study were highly effective in separating the 60 PVA inbred lines into heterotic groups. The PVA enriched maize inbred lines were classified into two heterotic groups based on both PVA content and grain yield and the two grouping methods agreed in classifying at least 40% of the inbred lines into the same heterotic groups. Also, the DArTag SNP markers showed high level of genetic diversity among the PVA enriched maize inbred lines and separated them into three clusters, which were consistent with three clustering methods. Even though both PVA and yield-based heterotic grouping of the inbred lines differed from their clusters defined by the DArTag SNP markers, the presence of the three marker-based clusters within each heterotic group can help in selecting PVA enriched maize inbred lines with diverse genetic backgrounds as parents for developing productive hybrids with high PVA content and for generating source populations to develop more vigorous maize inbred lines with much higher concentrations of PVA.

## Materials and methods

### Plant material and experimental design

Sixty PVA enriched maize inbred lines developed in the Maize Improvement Program of IITA and two inbred testers, (KU1414-SR/CI7/KU1414-SR)-63-B*6 (T1) with mean PVA concentration of 25 µg/g and 9450xKI21-7-3-1-2-5-B*7 (T2), with mean PVA concentration of 14.4 µg/g, were used in this study (Table S4). The PVA enriched maize inbred lines were developed by crossing elite maize inbred lines with intermediate levels of PVA with either elite PVA inbred lines or exotic tropical orange inbred lines [12]. The inbred lines are at S6 to S8 stage of inbreeding. The 60 inbred lines were crossed to the two testers using a line × tester mating design to form 120 testcrosses during the dry seasons (December 2019 to April 2020 and December 2020 to April 2021) at IITA’s research field, Ibadan (Table 3), Nigeria. The 120 testcrosses, the hybrid produced from a cross between the two testers and three commercial hybrid checks, Ife Hybrid-3, Ife Hybrid-4, and Oba Super 2 were evaluated at Ikenne, Saminaka, Zaria and Mokwa in Nigeria (Table 3) in 2020 and 2021. Ikenne is located in the rainforest ecology, while Mokwa, Zaria and Saminaka are located in the moist savannas.

**Table 3:** Description of the study locations

Location	GPS coordinates	Altitude (masl)	Ecology
Ibadan	3°54′ E, 7°29′ N	190	rainforest
Ikenne	3°42´ E, 6°54´ N	60	rainforest
Saminaka	8°39´ E, 10°34´ N	760	moist savannas
Zaria	7°45´ E, 11°8´ N	622	moist savannas
Mokwa	5°4´ E, 9°18´ N	457	moist savannas

The trial was arranged in a 31 × 4 alpha-lattice design with two replications. Plots consisted of single rows, each 5 m long, with plant-to-plant spacing of 0.25 m within rows, and 0.75 m between rows. Two seeds were planted per hill and thinned to one plant per hill after emergence to obtain a population density of 53,000 plants ha^− 1^. The fertilizer NPK 15:15:15 was applied at the rate of 60 kg N ha^− 1^, 60 kg P ha^− 1^ and 60 kg K ha^− 1^ at planting. Urea (46-0-0) was also applied at the rate of 30 kg N ha^− 1^ 4 weeks after planting. Herbicides (Primextra and Gramazone) were applied two days after planting as recommended for optimum maize production to control weeds. In addition, fall armyworm (FAW) was controlled by spraying the field with pesticide (caterpillar force), starting at three weeks after planting, then weekly till the crop attained horticultural maturity.

### Agronomic Data Collection

Plant height (PHT), ear height (EHT), days to anthesis (DYANTH), days to silking (DYSK), ear aspect (EASP), plant aspect (PASP), husk cover (HUSK), grain weight and percentage grain moisture content at harvest were recorded from the testcross trial. The measurement procedure for each trait was described by Maazou et al. [15]. The grain weight and moisture content were used to compute grain yield adjusted to 15% moisture.

### Carotenoid analysis

Every year, grain samples were taken from a composite grain of five self-pollinated ears in each plot at Ikenne and Saminaka for carotenoid analysis two to three weeks after harvest. Carotenoids were extracted from maize kernels and quantified by High-performance Liquid Chromatography (HPLC) (Water Corporation, Milford, MA, USA) at the Food and Nutrition Laboratory of IITA. The extraction protocol and carotenoid analysis used was based on the method described by Maazou et al. [12].

### DArTag genotyping

Leaf samples were collected from 10 seedlings of each inbred line and the testers three weeks after planting. The leaves were freeze-dried using Labconco Freezone 2.5 L system lyophilizer (Marshall Scientific, USA) and sent to the Diversity Arrays facility, Canberra, Australia [19] for DNA extraction and targeted genotyping with a proprietary maize SNP DArTag assay [20]. DArTag is a genotyping technology that amplifies selected SNPs discovered by DArTag [21] and genotyping by sequencing methods. The DArTag genotyping procedure was described by Maazou et al. [22].

### Data Analysis

For the field trials, each location-year combination was considered an environment. Using the line × tester procedure of Singh and Chaudhary [23], combined analysis of variance (ANOVA) was performed with Proc mixed procedure in SAS version 9.4 [24]. Hybrids were considered of fixed effects, while environment, replication (environment), block (replication × environment), environment × hybrid were considered as random effects in the linear model. After exclusion of the checks, the GCA and SCA effects of the parental inbred lines and the variance components for each trait were calculated with Analysis of Genetic Design (AGD-R, V.5.0) [25] as follows:

GCA = Line mean (X._j_) – Overall mean (X.)

SCA = Cross mean (X_ij_) – Line mean (X._j_) – Tester mean (X_i_.) + Overall mean (X.)

Restricted Maximum Likelihood Method (REML) was used to estimate the variance components [25].

PVA-based heterotic grouping of the inbred lines was performed based on their SCA effects and testcross mean PVA content following the method suggested by Menkir et al. [3]. Any inbred line that had a positive SCA with T1 but negative SCA with T2, and testcross mean PVA content not significantly different or greater than the mean PVA content of T1 × T2 was classified into the heterotic group B (HGB). Likewise, any inbred line with positive SCA with T2 but negative SCA with T1, and testcross mean PVA content not significantly different or greater than the mean PVA content of T1 × T2 was classified into the heterotic group A (HGA). A similar approach was used to classify the inbred lines into heterotic groups based on their SCA effects and testcross mean grain yields.

The PVA inbred lines were also grouped using the HSGCA values calculated based on the formula described by Fan et al. [2, 5] as follows:

### HSGCA = GCA + SCA

Inbred lines with positive HSGCA effects with T1 were assigned to HGB, whereas inbred lines with positive HSGCA effects with T2 were assigned to HGA. When an inbred line had either negative or positive HSGCA with both testers, we kept the inbred line with the heterotic group where it showed the smallest positive or the largest negative HSGCA value [22].

Spearman correlation analysis the PVA yield-based heterotic grouping methods was carried out using CORR procedure in SAS version 9.4 [24] to establish the concordance between the grouping methods .

A total of 3,305 SNPs were obtained from the DArT genotyping. PowerMarker version 3.25 [26] was used to filter out markers with > 10% missing data, major allele frequency (MAF) > 95%, and heterozygosity > 20% [18]. Finally, 1879 markers were retained for computing MAF, polymorphic information content (PIC), gene diversity, and heterozygosity with PowerMarker version 3.25 [26]. The 1879 markers were analyzed with the STRUCTURE version 2.3.4 software [27] which implements a Bayesian Markov chain Monte Carlo (MCMC) clustering procedure. The ADMIXTURE method with number of sup-groups (K) varying from 1 to 10 with 10 replications were used. Each replication was run with no prior information on the origin of individuals and iterations and burn-ins set to 10,000. The Evanno transformation method [28] was used to determine the most appropriate K-value within the PVA enriched maize inbred lines by implementing the structure results in Structure Harvester [29]. Inbred lines with membership probabilities equal to or greater than 60% were assigned to sub-groups while inbred lines with less than 60% membership probability were assigned to the mixed group [30].

PLINK [31] was used to calculate the pairwise genetic distance (identity-by-state, IBS) matrix among the inbred lines for the hierarchical cluster analysis. The IBS matrix was then used to build a Ward’s minimum variance hierarchical cluster dendrogram using the Analyses of Phylogenetics and Evolution (ape) package [32] implemented in R [33]. Principal Component Analysis (PCA) was also carried out in Tassel [34] to visualize the pattern of genetic dissimilarities within and between sub-groups.

### Electronic supplementary material

Below is the link to the electronic supplementary material.

**Additional file 1: Table S1**. Provitamin A-based classification of 60 PVA enriched maize inbred lines into heterotic groups.

**Additional file 2: Table S2**. Yield-based classification of 60 PVA enriched maize inbred lines into heterotic groups.

**Additional file 3: Table S3**. Provitamin A and yield-based separation of the lines into heterotic groups coupled with marker-based grouping of the PVA enriched maize inbred lines into clusters.

**Additional file 4: Fig. S1**. Summary statistics of 1879 markers used to assess the genetic diversity among the inbred lines.

**Additional file 5: Fig. S2**. Determination of the most appropriate K-value in structure analysis using Evanno’s Delta K.

**Additional file 6: table S5.** Genotypic data for maize Provitamin A inbred lines for diversity assessment and heterotic grouping.

**Additional file 7: table S4**. Maize inbred lines used in the present study.

## Data Availability

The datasets supporting the conclusions of this article are available in the manuscript and its additional files.
