# Supplementary material for: Heterotic grouping of provitamin A-enriched maize inbred lines for increased provitamin A content in hybrids
Source: BMC Genom Data. 2023 Sep 27;24:57. doi: 10.1186/s12863-023-01156-z (PMC10537512; doi:10.1186/s12863-023-01156-z)
Supplement: Supplementary file 4 — Additional file 4: Fig. S1. Summary statistics of 1879 markers used to assess the genetic diversity among the inbred lines. [file 12863_2023_1156_MOESM4_ESM.pdf]

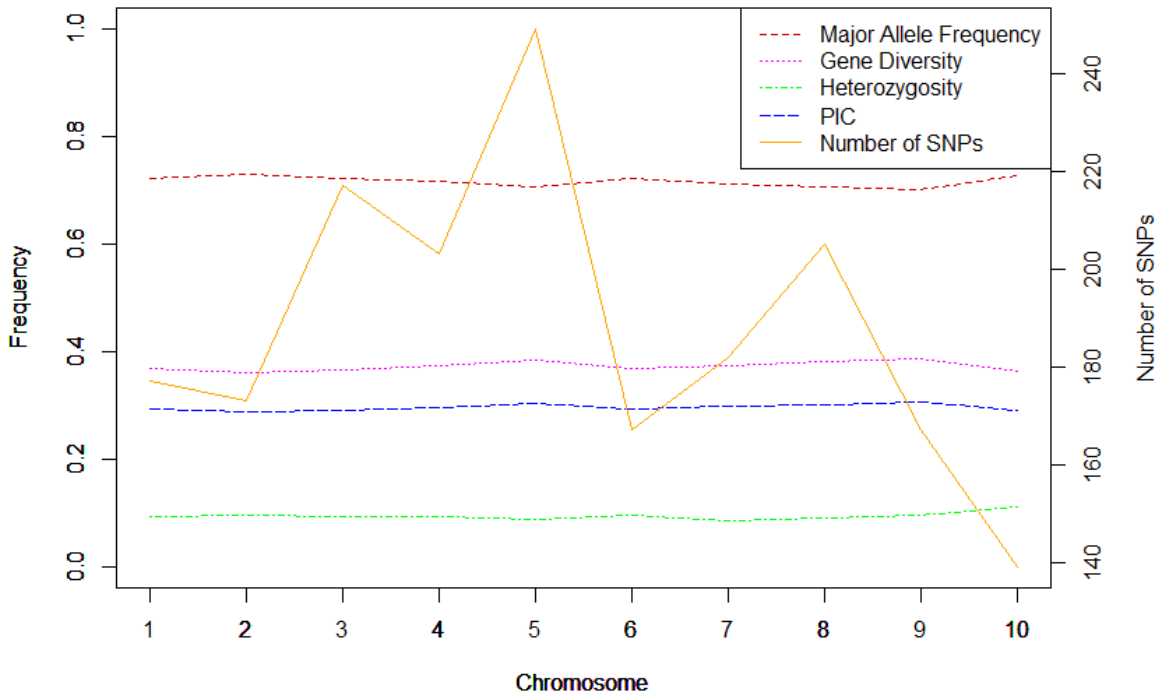

Fig S1. Summary statistics of 1879 markers used to assess the genetic diversity among the inbred lines
