# Supplementary material for: Heterotic grouping of provitamin A-enriched maize inbred lines for increased provitamin A content in hybrids
Source: BMC Genom Data. 2023 Sep 27;24:57. doi: 10.1186/s12863-023-01156-z (PMC10537512; doi:10.1186/s12863-023-01156-z)
Supplement: Supplementary file 5 — Additional file 5: Fig. S2. Determination of the most appropriate K-value in structure analysis using Evanno’s Delta K. [file 12863_2023_1156_MOESM5_ESM.pdf]

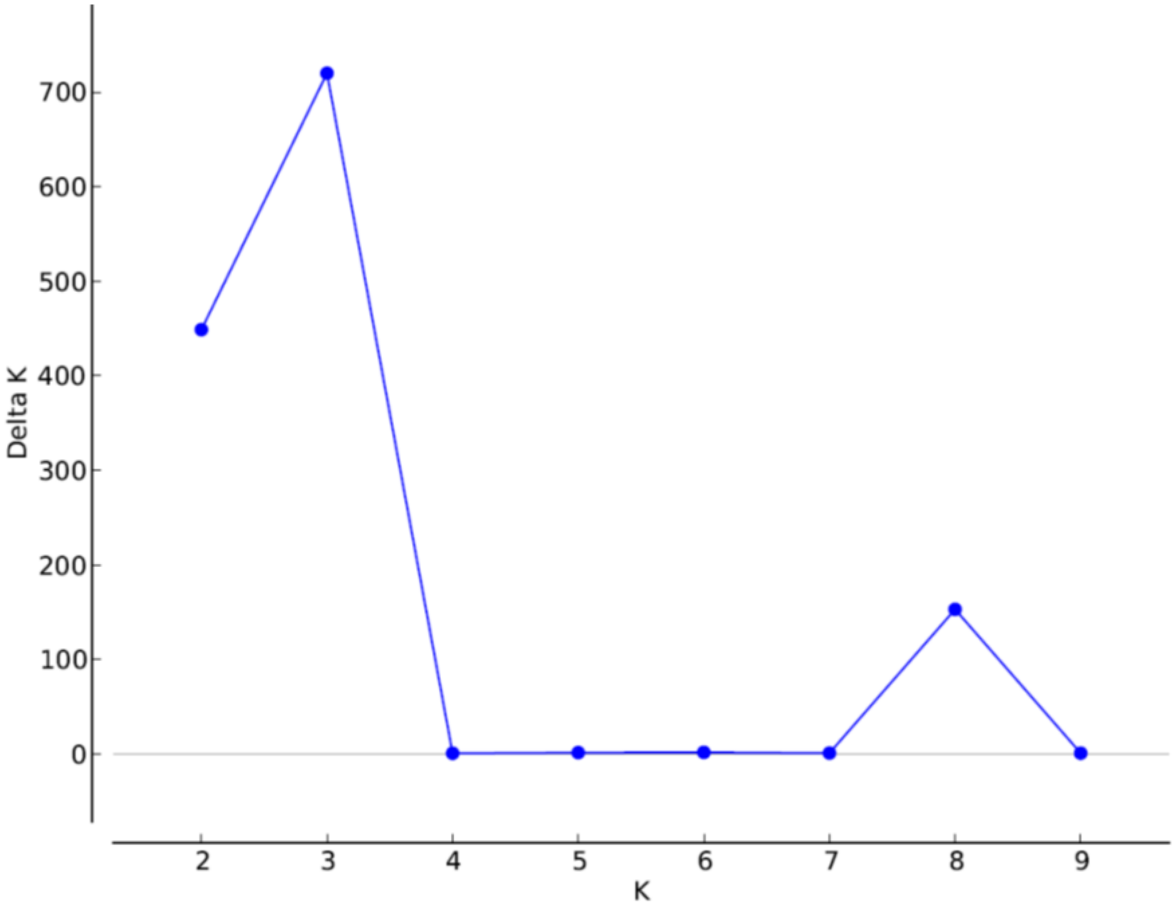

Fig S2. Determination of the most appropriate K-value in structure analysis using Evanno's Delta K.
